# Modification of Bentonite with Cationic and Nonionic Surfactants: Structural and Textural Features

**DOI:** 10.3390/ma12223772

**Published:** 2019-11-17

**Authors:** Magdalena Andrunik, Tomasz Bajda

**Affiliations:** AGH University of Science and Technology, Faculty of Geology, Geophysics and Environmental Protection, al. Mickiewicza 30, 30-059 Krakow, Poland; bajda@agh.edu.pl

**Keywords:** TX100, montmorillonite, HDTMA, adsorption

## Abstract

Surfactant-modified clay minerals are known for their good sorption properties of both organic and inorganic compounds from aqueous solutions. However, the current knowledge regarding the effect of both cationic and nonionic surfactants on the properties of bentonite is still insufficient. Bentonite, with montmorillonite as the base clay, was modified with hexadecethyltrimethylammonium bromide (a cationic surfactant) in the amount of 1.0 cation exchange capacity (CEC) of bentonite and varying concentrations of *t*-octylphenoxypolyethoxyethanol (Triton X-100, a nonionic surfactant). We aimed to improve the understanding of the effect of nonionic and cationic surfactants on clay minerals. The modified bentonites were characterized by X-ray diffraction (XRD), thermogravimetric analysis/differential thermal analysis (TG/DTA), Fourier transform infrared spectrometry (FTIR), field emission scanning electron microscopy (SEM) and specific surface area and pore volume (BET). According to our results, the presence of a cationic surfactant significantly increased the amount of the adsorbed nonionic surfactant. Moreover, an increase in the concentration of nonionic surfactants is also associated with an increase in the effectiveness of the modification process. Our results indicate that the amount of nonionic surfactant used has a significant effect on the properties of the obtained hybrid material. Modification of bentonite with a nonionic surfactant did not cause an expansion of the interlayer space of smectite, regardless of the presence of a cationic surfactant. The modification process was found to significantly decrease the specific surface area of bentonite. Improvement of hydrophobic properties and thermal stability was also observed.

## 1. Introduction

Bentonite is an aluminum phyllosilicate clay consisting primarily of montmorillonite. It is obtained by open pit mining and used in various applications such as in the production of ceramics, adhesives, catalysts, desiccants, cosmetics, and has pharmaceutical applications. In geotechnical engineering, bentonite has found various applications, especially in pollution control [1,2,3,4]. It is the most extensively used clay due to its abundance, low cost, and ease of availability [5,6]. Montmorillonite—the clay mineral—belongs to the smectite group of minerals and is the most common member of the clay minerals. It has a 2:1-type layer silicate structure, which has excellent sorption capacity for many heavy metals [7,8,9]. A characteristic feature of the smectite group is that water and other polar molecules can cause the structure to expand in the direction which is normal to the basal plane, by entering between the unit layers. The minerals belonging to this group have an overall negative surface charge; thus, cations can be easily adsorbed onto their surface [10]. This characteristic property is due to the presence of isomorphic substitutions in their structure.

The negative surface charge of natural bentonite limits its sorption capacity with respect to anionic and nonionic contaminants. The surface charge can be changed by performing the modification process with organic surfactants [11,12,13,14]. A previous study indicates that the first hybrid inorganic–organic composites—clay minerals modified with quaternary alkylammonium ions—were developed to improve the compatibility of clay minerals with less polar matrices [15]. Experiments proved that basal spacing increases and the specific surface area decreases with an increasing concentration of surfactant [16,17]. Such modifications also transform the surface of the silicate from being hydrophilic to being organophilic and reduce the swelling behavior of the modified clay [18,19,20]. Surfactant-modified clay mineral—the organo-clay—can create an adsorption zone to intercept contaminants; thus, it can be used as a barrier for in situ remediation of contaminated water and soils [21,22].

Although there is a large amount of data on the modification of montmorillonite with different surfactants, information regarding the effect of a nonionic surfactant alone or the combination of both cationic and nonionic surfactants on the properties of bentonite is limited [23]. The modification of clay minerals with cationic surfactants is well-known and well-described in the literature. Cationic surfactant-based sorbents are extensively used in environmental remediation [24,25]. Nevertheless, they are considered to be toxic and therefore might not be suitable for use in certain large scale environmental and biomedical applications [26]. Nonionic surfactant-based organo-bentonites are more chemically stable and less toxic than cationic surfactant-based organo-bentonites. Greater interlayer spacing can be achieved by hydrogen bonding between the oxide and the silicate surface [23,27,28,29]. The literature indicates that hexadecethyltrimethylammonium bromide (HDTMA-Br) is one of the most frequently used cationic surfactants in the modification of clay minerals [30,31,32]. HDTMA-modified clay minerals are commonly used in the process of environmental remediation, especially as sorbents of inorganic contaminants [8,33,34,35,36,37,38,39]. Nonionic surfactants are rarely used in the modification of clay minerals; nevertheless, the properties of such hybrid materials are highly promising [1,11,40,41,42,43,44,45,46,47]. In addition, *t*-octylphenoxypolyethoxyethanol (Triton X-100 (TX100)) is one of the most popular nonionic surfactants in terms of clay modification [11,48,49,50,51,52,53]; however, the interaction of bentonite with TX100 has not been studied extensively.

Therefore, in this study, we aimed to investigate the structural and textural features of bentonite modified with cationic and nonionic surfactants. Modification of clay minerals with a nonionic surfactant and with both cationic and nonionic surfactants has been previously reported; however, the proper understanding of the changes in structural and textural properties of the sorbents used is very crucial to determine the potential application of their hybrid materials. An additional challenge was to identify the most stable conditions that are suitable for the modification processes. In this study, we present the features of organically modified bentonite.

## 2. Materials and Methods

### 2.1. Materials

In this study, we used montmorillonite phase bentonite from Jelšovy Potok, Slovakia. Prior to the experiments, the samples were air-dried, sieved through a 2 mm sieve, and thoroughly crushed in the agate mortar. For all experiments, only the fraction containing a grain size of <0.2 mm was used. The cation exchange capacity (CEC) of the bentonite was 48 meq/100 g, and the chemical composition was as follows: SiO_2_ 71.2%, Al_2_O_3_ 16.4%, Fe_2_O_3_ 1.9%, CaO 1.5%, MgO 1.9%, Na_2_O 0.1%, K_2_O 0.9%, TiO_2_ 0.1%.

Hexadecethyltrimethylammonium bromide (HDTMA-Br) has a linear structure of 19 ethylenes, compensated with a Br^-^. Its molecular weight is 364.45 g/mol. The size of the HDTMA-Br molecule is 25 Å. The polymerization product of isooctylphenol and ethylene oxide, *t*-octylphenoxypolyethoxyethanol (Triton X-100; TX100), has an average of 9.5 ethylene oxide units per molecule of aromatic hydrocarbon lipophilic or hydrophobic groups. Its average molecular weight is 625 g/mol, while average length is 37 Å. All chemicals used were of analytical grade and were purchased from Sigma-Aldrich Co., Poznan, Poland.

### 2.2. Modification

Bentonite was modified with two organic salts: HDTMA-Br and TX100. The modification was performed in two independent ways:

1. Two-stage modification of bentonite with cationic and nonionic surfactants. First, the modification of bentonite by exchanging naturally occurring cations at the mineral ion exchange positions for organic cations of HDTMA was conducted. The samples were prepared by mixing 100 g of natural bentonite with 2000 g of the HDTMA solution at a concentration of 48 mmol (1.0 CEC). After 8 h of stirring at 60 °C, the samples were centrifuged for 10 min at 14,000 rpm, washed thrice with distilled water and once with hot ethanol, and then dried at 40 °C for 24 h. This procedure has been well documented and proven to be highly effective [36]. Then, the prepared material was modified using a nonionic surfactant (TX100). The samples were stirred with 2000 g of TX100 solution at a concentration range of 2–120 mmol/100 g at 40 °C. After 24 h of stirring, the samples were centrifuged for 10 min at 14,000 rpm and dried at 40 °C for 24 h.
2. Single-stage modification of bentonite by exclusively using a nonionic surfactant with different initial molar concentrations was conducted. The samples were stirred with 2000 g of TX100 solution at concentrations of 2–120 mmol/100 g at 40 °C. After 24 h of stirring, the samples were centrifuged for 10 min at 14,000 rpm and dried at 40 °C for 24 h.

To estimate the amount of HDTMA adsorbed during the modification process, we measured the content of nitrogen (N), carbon (C), and hydrogen (H) in bentonite before and after HDTMA treatment. We used an automatic analyzer for the measurements (CHNS Vario EL III, Elementar Analysensysteme GmbH, Langenselbold, Germany). The amount of TX100 adsorbed was estimated by the procedure described by Garewal [54]. The measurements were obtained using a Hitachi U-1800 ultraviolet-visible (UV-Vis) spectrophotometer (Hitachi—Science and Technology, Wokingham, UK).

The analysis of mineralogical and physical properties of the obtained sorbents have been conducted only for modifications containing 2 mmol, 48 mmol, and 120 mmol of TX100/100 g. Samples of modified bentonite were named as follows:

- Natural bentonite: BENT
- Bentonite modified with HDTMA only: HD-B
- Bentonite modified with HDTMA and TX100 (2 mmol of TX100/100 g): HD-TX-2
- Bentonite modified with HDTMA and TX100 (48 mmol of TX100/100 g): HD-TX-48
- Bentonite modified with HDTMA and TX100 (120 mmol of TX100/100 g): HD-TX-120
- Bentonite modified with TX100 alone (2 mmol of TX100/100 g): TX-2
- Bentonite modified with TX100 alone (48 mmol of TX100/100 g): TX-48
- Bentonite modified with TX100 alone (120 mmol of TX100/100 g): TX-120

### 2.3. Methods

The mineral composition of the investigated samples was obtained via powder X-ray diffraction (XRD) by using a SmartLab RIGAKU diffractometer (RIGAKU, Tokyo, Japan) with graphite-monochromatized Cu_Kα_ radiation operating at 9 kV. The measurements were conducted at 2–75° 2θ (depending the measurement) with a measuring step of 0.05 2θ/s. The XRD patterns were interpreted using the XRayan program (Version 4.2.2, “KOMA,” Warsaw, Poland). The Fourier transform infrared (FTIR) spectra were recorded with a Nicolet 6700 spectrometer (Fishers, Waltham, MA, USA) using the drift technique (3 wt% sample/KBr) with 64 scans at 4 cm^−1^ resolution in the 4000–400 cm^−1^ region. Morphology observations of air-dried, uncoated samples were analyzed by scanning electron microscopy (SEM) using a variable pressure field emission scanning electron microscope (FEI Quanta 200, FEI, Graz, Austria). Thermogravimetric analysis/differential thermal analysis (TG/DTA) coupled with the measurement of the composition of evolved gases was performed using a Netzsch STA 449 F3 Jupiter apparatus (Netzsch, Chennai, India). Samples were heated at a temperature of 30–1000 °C (heating rate: 10 °C/min). The analyses were conducted in combustion conditions. The specific surface area and porosity were determined from N_2_ gas adsorption/desorption isotherms at −196 °C using an ASAP 2020 apparatus (Micromeritics, Norcross, GA, USA). Prior to the measurements, the samples were outgassed for 12 h at 105 °C. Based on the data obtained from N_2_ isotherms, specific surface area (*S_BET_*) was calculated by applying the Brunauer–Emmett–Teller (BET) equation [55]. The total pore volume was calculated from the amount of N_2_ adsorbed at a relative vapor pressure (p/p^0^) of approximately 0.99. The volume of the micropores was calculated by applying the Dubinin–Radushkevich method [56]. The mesopore volume was determined from the adsorption branch of the isotherms by using the Barrett–Joyner–Halenda (BJH) method [57] in the mesopore range proposed by Dubinin [56]. The macropore volume (Vmac) was calculated by using the following equation:

(1) $$V_{\mathrm{mac}}=V_{\mathrm{tot}}^{0.99}-\left(V_{\mathrm{mic}}^{\mathrm{DR}}+V_{\mathrm{mes}}^{\mathrm{BJH}}\right)$$

where $V_{\mathrm{mic}}^{\mathrm{DR}}$ is the volume of micropores and $V_{\mathrm{mes}}^{\mathrm{BJH}}$ is the volume of mesopores.

## 3. Results and Discussion

### 3.1. Modification

Table 1 presents the quantity of the nonionic surfactant adsorbed by the sorbent by using both methods of modification. The results of the experiment performed to determine the effect of the concentration of the surfactant on the modification of natural bentonite and HDTMA–bentonite clearly show that an increase in the concentration of nonionic surfactant in the solution is associated with an increase in the effectiveness of the modification process. Regardless of the modified material, the higher the initial concentration of TX100, the higher sorption effectiveness. In the case of natural bentonite, the effectiveness of modification did not exceed 40 mmol/100 g. However, the cationic surfactant significantly increased the amount of TX100 adsorbed onto the surface of bentonite, especially at higher initial concentrations.

Our results indicate that HDTMA molecules enhance the adsorption of TX100 molecules onto the surface of bentonite, which was negatively charged due to isomorphic substitution. Therefore, HDTMA cations can be adsorbed onto the surface of bentonite through electrostatic interactions and via replacement of the inorganic exchangeable cations with layer silicate clays [11,58]. Pre-attached HDTMA enables the formation of mixed aggregates with TX100 through chain–chain interactions. Similar processes have been reported by Zhang et al. [11]. However, the modification of bentonite with TX100 alone is caused by the occurrence of hydrogen bonds; therefore, the amount of nonionic surfactant adsorbed onto the surface of natural bentonite is lower, especially at an initial concentration of TX100 higher than 48 mmol/100 g [11,59].

### 3.2. XRD

**Figure 1:**
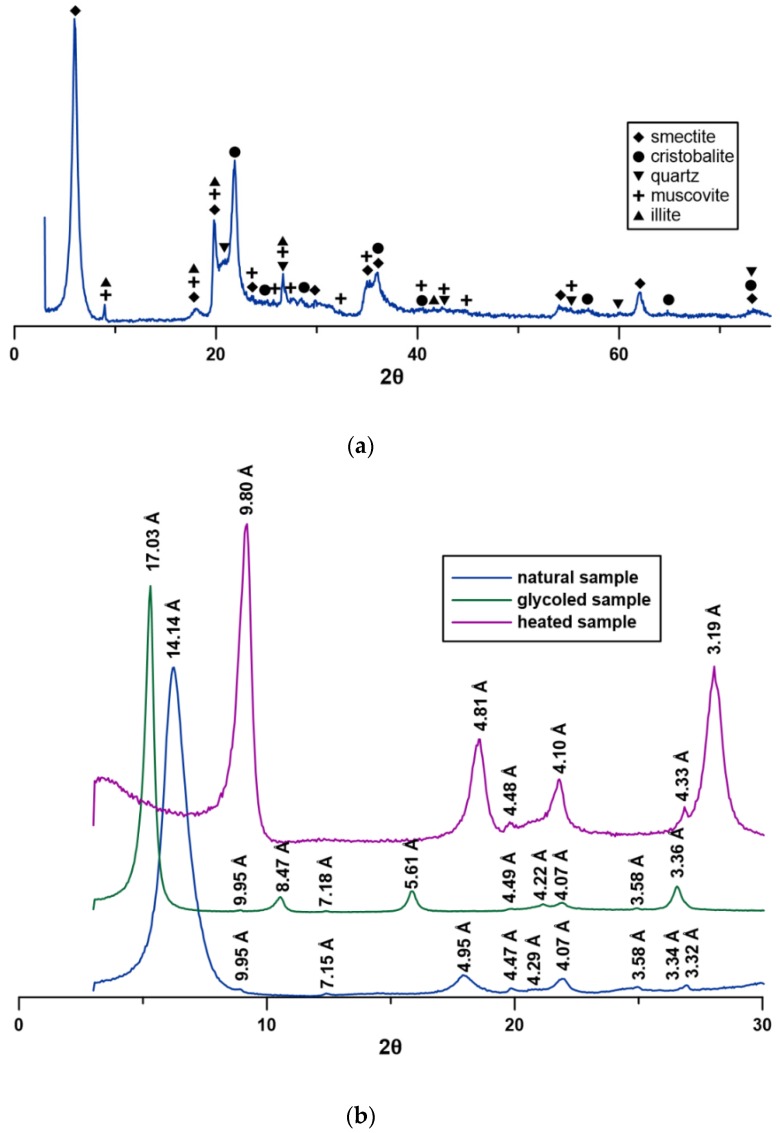
X-ray diffraction patterns of bentonite. (**a**) Mineralogical composition, (**b**) oriented samples.

Figure 1 shows the results of the XRD spectra of natural bentonite. The studied bentonite consists mostly of clay minerals (smectite, illite, and cristobalite) with admixtures of muscovite and quartz (Figure 1a). The position of the peaks on oriented samples (Figure 1b) allows us to distinguish main clay minerals in the bentonite sample. The XRD analysis of the air-dried sample gave a very strong 001 diffraction peak at 14.14 Å which, in the glycol-treated sample, shifts to 17.03 Å. This indicates that Ca^2+^ is the predominant cation in the smectite interlayer positions [60]. Final identification was accomplished by drying the sample at 300 °C. This treatment shifted the 001 diffraction peak to 9.80 Å, indicative of montmorillonite collapse. Small quantities of illite (9.95 Å, 4.95 Å, and 3.31 Å), kaolinite (7.15 Å and 3.58 Å), and cristobalite (4.07 Å) were also present (Figure 1b).

**Figure 2:**
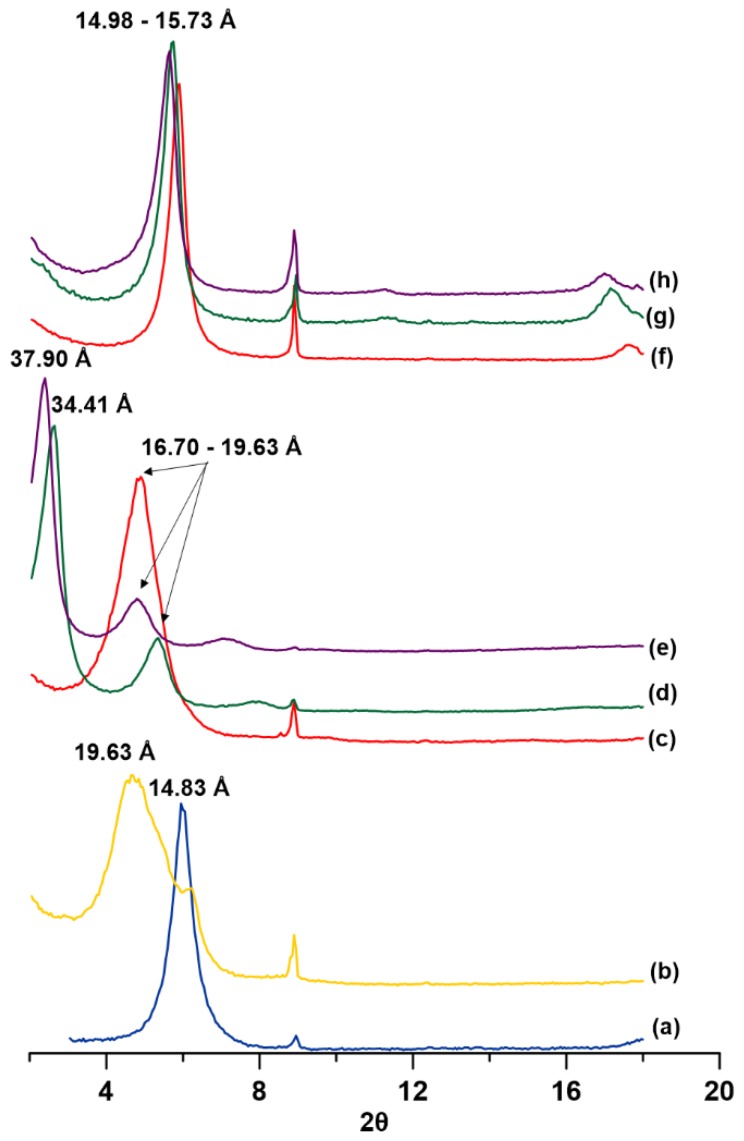
X-ray diffraction patterns of: (**a**) natural bentonite (BENT); (**b**) bentonite modified with HDTMA only (HD-B); (**c**) bentonite modified with HDTMA and TX100 (2 mmol of TX100/100 g) (HD-TX-2); (**d**) bentonite modified with HDTMA and TX100 (48 mmol of TX100/100 g) (HD-TX-48); (**e**) bentonite modified with HDTMA and TX100 (120 mmol of TX100/100 g) (HD-TX-120); (**f**) bentonite modified with TX100 alone (2 mmol of TX100/100 g) (TX-2); (**g**) bentonite modified with TX100 alone (48 mmol of TX100/100 g) (TX-48); (**h**) bentonite modified with TX100 alone (120 mmol of TX100/100 g) (TX-120).

Figure 2 shows the results of XRD patterns of bentonite before and after modification with organic compounds. The main peak (001) from smectite was observed to shift toward higher interlayer distances. The intercalation of large surfactant molecules resulted in an increase in the interlayer space. The modification of bentonite with HDTMA increased the distance to 19.63 Å (Figure 2b). The arrangement of the cationic surfactant based on alkylammonium ions in the interlayer space of smectites is known to move gradually from a monolayer to a bilayer, and then to a pseudo-trimolecular layer and then to a paraffin arrangement. The arrangement depends on the concentration of the surfactant and the layer charge density within the clay. For example, HD-B basal spacing increased to 19.63 Å which corresponds to bilayer coverage [1,61,62,63].

Modification of HDTMA–bentonite with TX100 did not cause further expansion of the interlayer space in smectite if the amount of nonionic surfactant is small (sample HD-TX-2; Figure 2c), however, except for the samples HD-TX-48 and HD-TX-120 (Figure 2d–e), a new strong peak at 34.41 Å and 37.9 Å were visible in the XRD spectrum. According to the literature, the peaks at those positions suggest that TX100 molecules intercalated within the interlayer spaces of HDTMA–bentonite [64]. At low initial concentrations of TX100 (2 mmol/100 g of bentonite), expansion of the interlayer space was small, although the UV-Vis spectra clearly show that TX100 molecules were adsorbed onto the HDTMA–bentonite clay. This suggests that a nonionic surfactant is more easily adsorbed onto the surface of HDTMA–bentonite than that of the interlayer space. Cointercalaction of cationic and nonionic surfactants at higher concentrations of TX100 (48 mmol and 120 mmol/100 g of bentonite) is probably the result of saturation of the active centers on the surface of the bentonite [64].

The modification of bentonite with TX100 alone (Figure 2f–h) caused a slight expansion of the interlayer space of smectite (14.98 Å–15.73 Å). This could be due to the fact that the molecule of TX100 is too big to intercalate between the interlayer space; therefore, expansion of the interlayer space was not significant, and the modification of bentonite with TX100 was seen mostly on the surface. This result is consistent with the results reported by Breen et al. [49], who did not observe a significant change in basal spacing of montmorillonite after absorption of TX100.

### 3.3. FTIR

**Figure 3:**
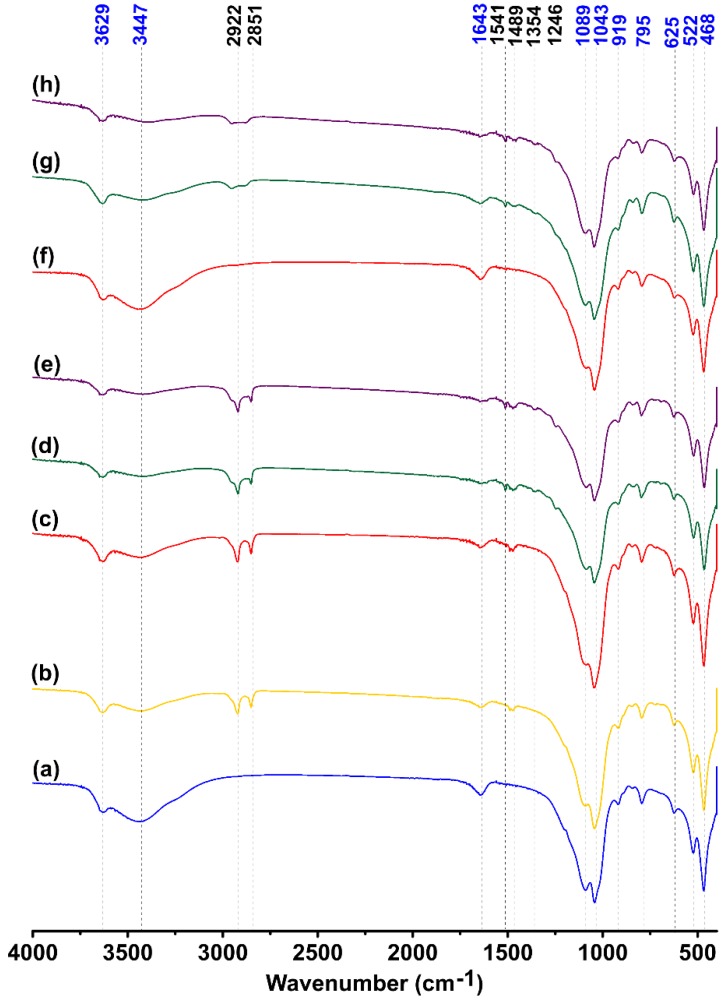
Fourier transform infrared (FTIR) spectra of: (**a**) BENT, (**b**) HD-B, (**c**) HD-TX-2, (**d**) HD-TX-48, (**e**) HD-TX-120, (**f**) TX-2, (**g**) TX-48, and (**h**) TX-120 (blue values—bands derived from natural bentonite; black values—bands that appear after adsorption of the surfactants).

Figure 3 shows the FTIR spectrum of natural and modified bentonite samples. The absorption band visible at the 3629 cm^−1^ region is due to the stretching vibrations of structural OH groups of bentonite, whereas the bands visible at the 3447 cm^−1^ and 1643 cm^−1^ regions correspond with H–O–H vibrations in water (tensile vibrations and deformation vibrations, accordingly). Major bands in the 1089–468 cm^−1^ region are ascribed to bond vibrations in the structure of the examined mineral and are associated with the vibration of Si–O–Si (1089 cm^−1^, 1043 cm^−1^, and 468 cm^−1^) and Si–O–Al bridges (522 cm^−1^). The absorption band at the 919 cm^−1^ region corresponds to the deformation vibrations of the Al–Al–O bond, and the bands at the 795 cm^−1^ and 625 cm^−1^ regions suggest the presence of stretching vibrations of Si–O bonds. The absorption band at the 625 cm^−1^ region is derived from both the deformation and bending modes of Si–O and Al–O bonds. A sharp absorption band at the 795 cm^−1^ region indicates quartz admixture in the sample.

The FTIR spectrum of HD-B shows bands in the region ranging from 3000–2800 cm^−1^ (Figure 3b), which correspond to the anti-symmetric and symmetric tensile vibrations of methylene groups (–CH_2_) of the hydrocarbon tails of the surfactants [65,66], whereas a small band at the 1489 cm^−1^ region is ascribed to the bending vibrations of C–H [67]. The occurrence of the bands corresponding to the adsorbed water indicate that not all the water has been replaced by surfactant molecules. However, changes in the intensity of these bands suggest partial displacement of water molecules by the adsorbed surfactant [68].

Figure 3c–e presents the FTIR spectra of bentonite modified with both HDTMA and TX100. HD-TX-2 did not show any new bands compared to HD-B. However, HD-TX-48 and HD-TX-120 exhibited new bands at the 1541 cm^−1^, 1354 cm^−1^, and 1246 cm^−1^ regions in addition to the main bands visible in the spectrum of HD-B. These bands are derived from CH stretching and bending and CO and OH bending vibrations of TX100 molecules [68]. Moreover, absorption bands in the region ranging from 3629–3447 cm^−1^ and at 1643 cm^−1^ originated from water molecules, which coincides with the OH stretching and bending vibration of TX100. C–C bonds originating from phenyl rings should be visible around 1640 cm^−1^, which suggests that in samples containing TX100, coincidence between C–C and O–H_2_ bonds may occur [69]. HD-TX-2 does not show the presence of bands originating strictly from TX100 molecules; however, since coincidence between bands can occur, it cannot be excluded that the FTIR spectrum contains absorption bands that represent a small amount of TX100 adsorbed onto the surface of the sample.

Figure 3f–h presents the FTIR spectra of bentonite modified with TX100 alone. Compared to natural bentonite (Figure 3a), a few new bands are visible for all samples except for TX-2. This suggests that the amount of TX100 adsorbed by the sample is too small to be detected by this method. In the case of TX-48 and TX-120, the position of the new bands is similar to those that occur on the spectra of bentonite modified with both HDTMA and TX100—all bands characteristic for HDTMA can also be the evidence of the presence of TX100. The effectiveness of the modification of bentonite with TX100 is confirmed by the presence of the following weak absorption bands: 2922–2851 cm^−1^, which represent methylene groups (–CH_2_); 1541 cm^−1^, 1354 cm^−1^, and 1246 cm^−1^, which represent CH stretching and bending and CO and OH bending vibrations of TX100 molecules; and a band at 1643 cm^−1^ from C–C bonds of phenyl rings [68,69]. It confirms that the modification of the samples by TX100 has been achieved.

A decrease in the intensity of the bands which represent OH groups indicates a reduction in the interlayer water content. The reduction is primarily caused by the hydrophobization of the surface and replacement of water molecules with surfactant molecules [70,71]. Bands derived from nonionic surfactants are weak and did not occur in the case of HD-TX-2 and TX-2. In addition, peaks related to the presence of water molecules are stronger for HD-TX-48, HD-TX-120, TX-48 and TX-120. This confirms that at low concentrations of TX100, surface overlaying is insufficient to be detected. Moreover, bands that show the presence of surfactant molecules are weaker for bentonite modified with TX100 alone than that of the bands for bentonite modified with both HDTMA and TX100. This confirms the UV-Vis results, which indicates that the presence of HDTMA enhances the adsorption of TX100. Moreover, the occurrence of bands corresponding to the CO bending vibrations at 1354 cm^−1^ indicates a possible ion–dipole interaction between the surfactant and the silicate molecules through the OH group of TX100 and the water molecules coordinated to the exchangeable cations of clay minerals [68].

### 3.4. TG/DTA

**Figure 4:**
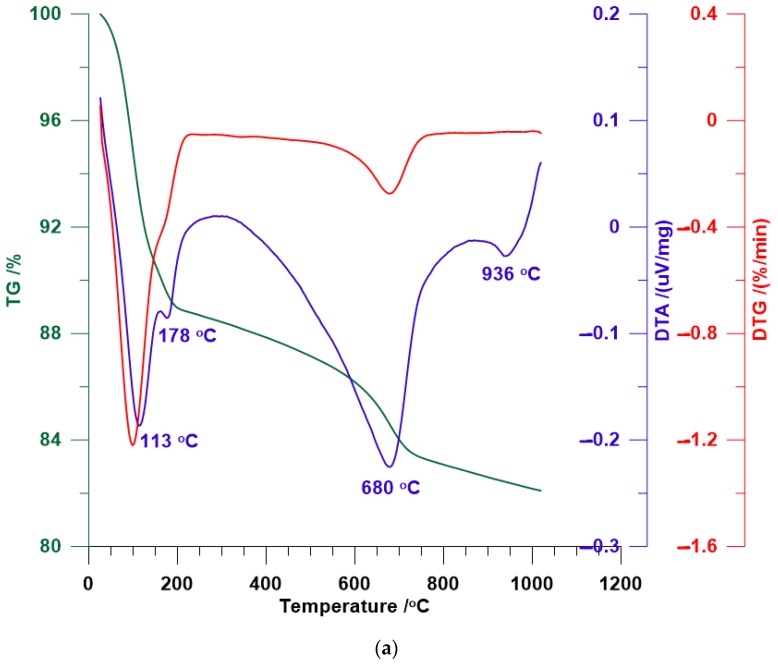

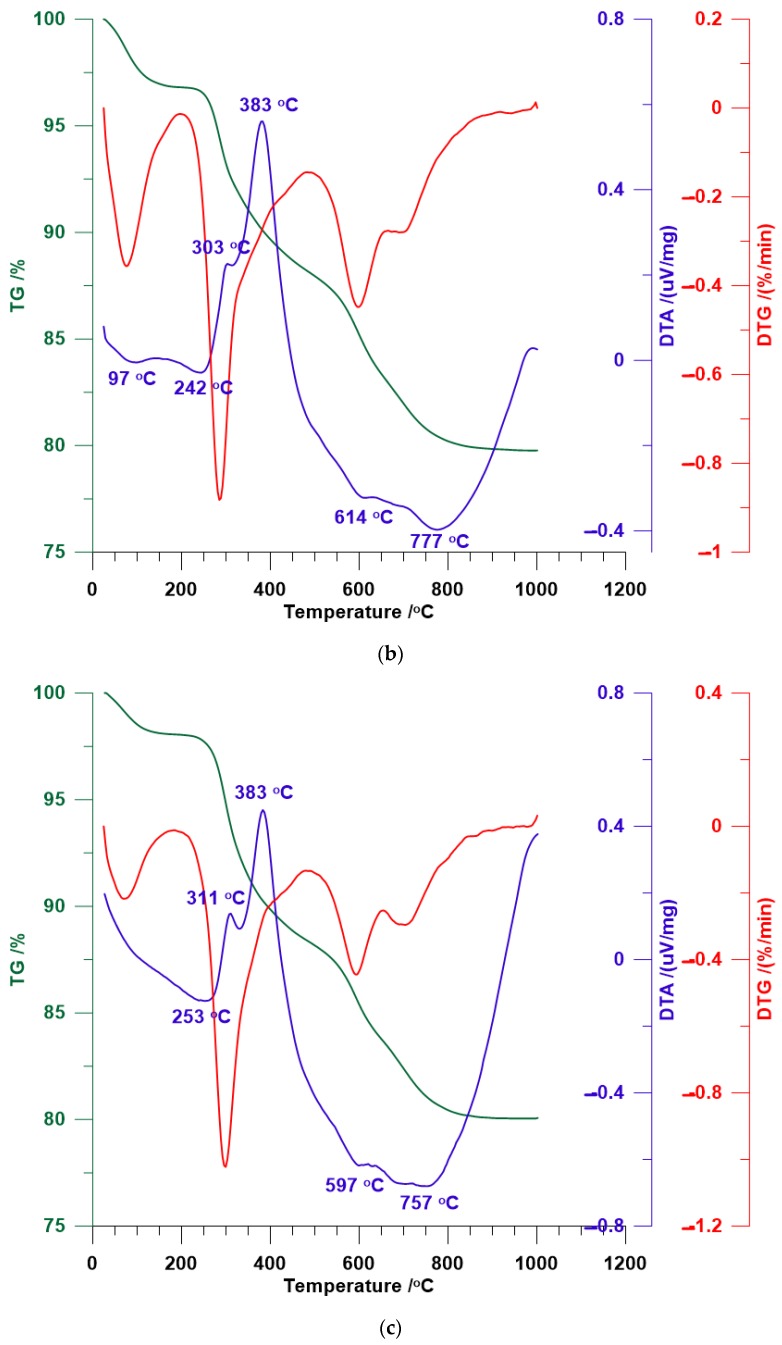

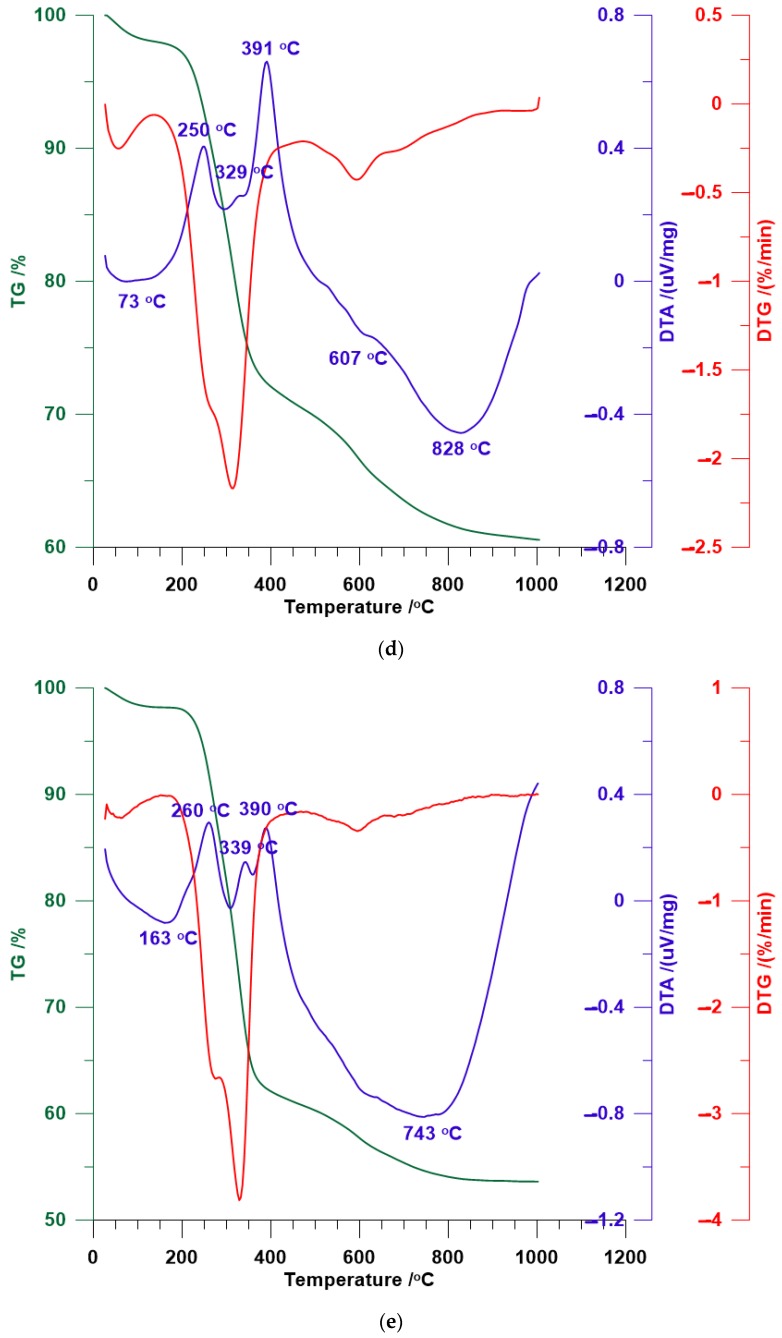

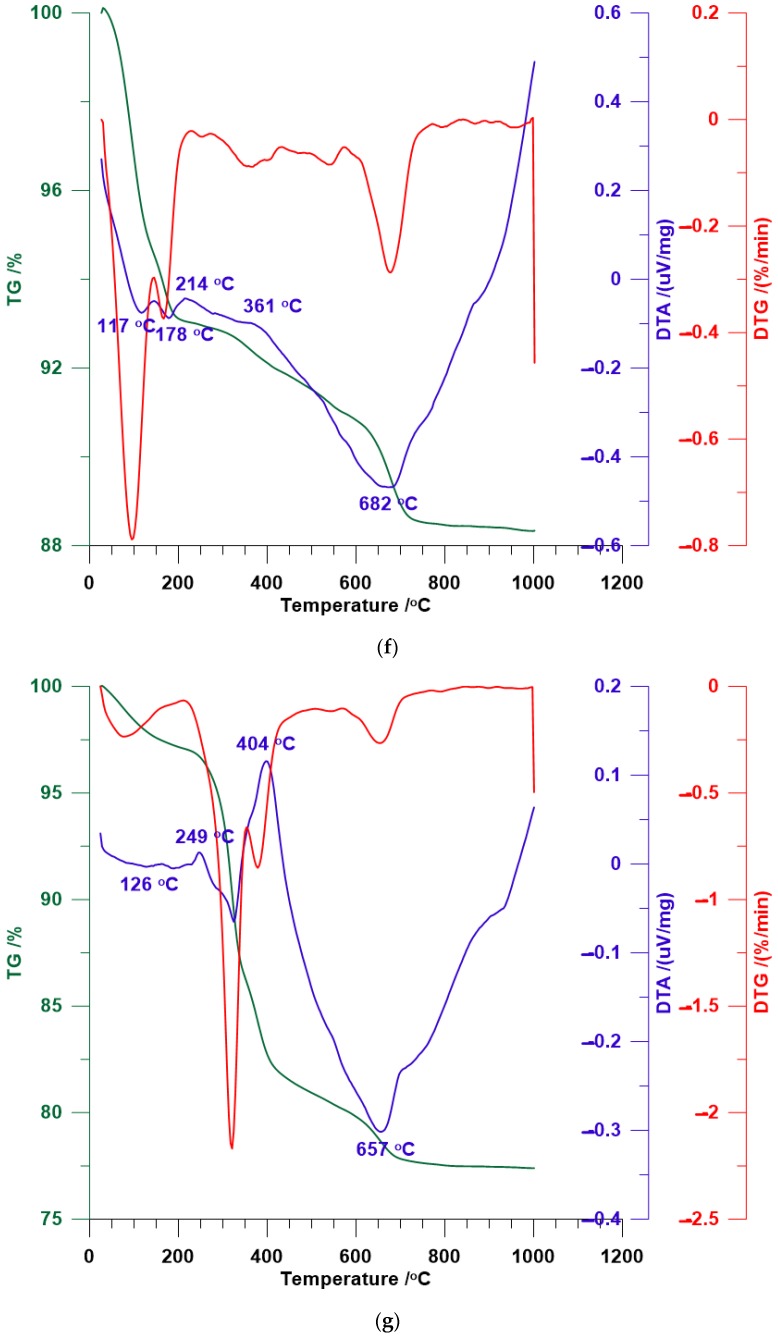

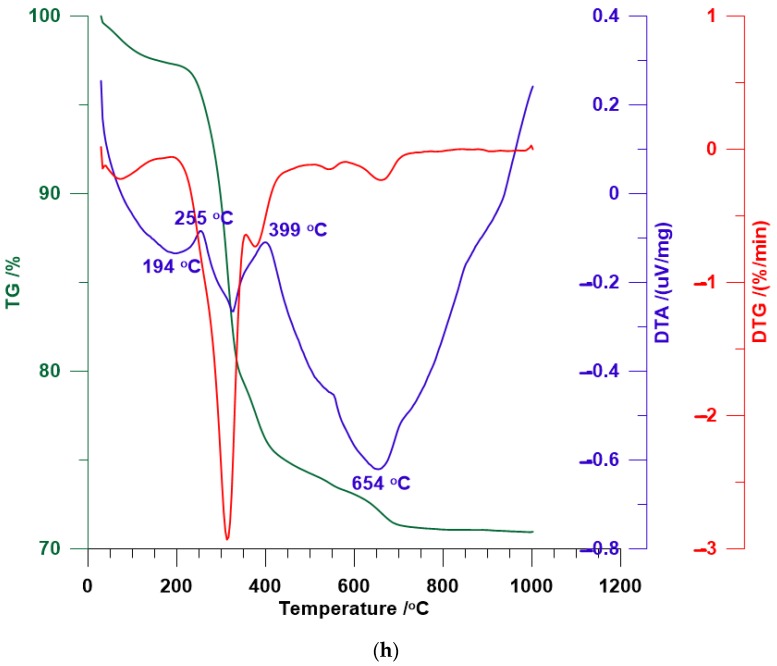
Thermal analysis thermogravimetric analysis/differential thermal analysis (TG/DTA) of: (**a**) BENT, (**b**) HD-B, (**c**) HD-TX-2, (**d**) HD-TX-48, (**e**) HD-TX-120, (**f**) TX-2, (**g**) TX-48, and (**h**) TX-120.

Figure 4 shows the results of thermal analysis of natural and modified bentonite. The DTA curve for BENT (Figure 4a) revealed several endothermic effects at temperatures of approximately 113 °C and 178 °C and also at higher temperatures of approximately 680 °C and 936 °C. The peak value at approximately 113 °C represents the removal of water molecules physically adsorbed onto the surface of bentonite, whereas the peak value at 178 °C reveals the removal of water molecules from interlayer space of montmorillonite—the primary component of bentonite. The shape and the position of the aforementioned peaks are the effects of dehydration of Ca-montmorillonite [72]. At 680 °C, the endothermic effect is associated with dehydroxylation of clay OH units and probable formation of an amorphous meta-montmorillonite phase, whereas the endothermic peak at 936 °C is responsible for the structural breakdown of bentonite [72]. TG analysis shows an 11% loss of mass, which might be due to the dehydration process that takes place at 100–200 °C, and the subsequent loss of mass (5–6%) at 600–1000 °C is probably associated with dehydroxylation and structural breakdown.

DTA curves of HD-B indicate the occurrence of several endothermic and exothermic effects (Figure 4b). At low temperatures (around 97 °C), the weak endothermic effect is associated with dehydration of bentonite. Exothermic effects in the temperature range of 303–383 °C are attributed to the combustion and loss of the surfactant molecules from the surface and from the interlayer space. HDTMA molecules are either decomposed or combusted at approximately 210 °C [73,74]. The fact that the surfactant molecules are not lost until almost 400 °C indicates that the surfactant molecules are strongly bonded in the interlayer of montmorillonite. At temperatures above 600 °C, the effects resulting from the dehydroxylation and breakdown of the mineral structure are predominantly observed. Temperatures of dehydroxylation and breakdown of the mineral structure are lower than for natural bentonite—this suggests bonding of the methyl groups of the surfactant with the siloxane layer [74]. The higher loss in total mass (20%) or HD-B compared to that of BENT is associated with decomposition of adsorbed surfactant.

Figure 4c–e shows the DTA curve for bentonite modified with HDTMA and TX100. The shapes of the curves are different depending on the amount of TX100 adsorbed by the bentonite. Pure TX100 is decomposed or combusted at a temperature of approximately 310 °C [68]. For HD-TX-2 (Figure 4c), the exothermic effects at a temperature of approximately 311 °C are slightly higher than that of HD-B. It can be explained by the decomposition or combustion of small amount of adsorbed TX100. For HD-TX-48 and HD-TX-120, new exothermic effects at temperatures of approximately 250–260 °C are visible, which is a result of melting of non- or weakly-adsorbed surfactant molecules and peaks at approximately 400 °C, which is responsible for the destruction of surfactant molecules [68]. It can explain the occurrence of two peaks which represent the destruction of surfactant molecules. Very broad effects at temperatures ranging from 600–900 °C originate from dehydroxylation and breakdown of the mineral structure.

Figure 4f–h presents the curves for bentonite modified with TX100 alone. At a low concentration of TX100 (Figure 4f), three small endothermic effects represent dehydration (approximately 117 °C and 178 °C) and dehydroxylation (approximately 682 °C) of clay minerals. The only evidence of TX100 adsorbed onto the surface of bentonite is very weak effects seen at temperatures of approximately 214 °C and 361 °C, which is related to the decomposition or combustion of small amounts of TX100. It is consistent with the results of XRD—for HD-TX-2, no significant changes are visible compared to natural bentonite. In the case of HD-TX-48 and HD-TX-120, peaks responsible for the dehydration are no longer visible; however, exothermic effects related to the decomposition or combustion of TX100 are stronger and shifted to higher temperatures (approximately 250 °C and 400 °C). The occurrence of these peaks can indirectly prove the effectiveness of the modification process. The interaction of surfactant molecules with the structure of the mineral minimizes the disintegration of the surfactant, which occurs at a temperature higher than what is required to disintegrate pure TX100. The decomposition of TX100 molecules, with 9.5 ethoxylate groups and one OH group, must consist of two steps: first, rupture of C–OH, the ethoxylate part and the C–C alkyl part and second, the process of combustion [68]. This was also observed in the case of other surfactants [75,76]. Moreover, the oxidation reaction of the organic material derived from a surfactant usually takes place in two steps. At temperatures ranging from 200–500 °C, the oxidation of organic hydrogen and formation of water and charcoal occurs, whereas at temperatures ranging from 400–750 °C, the oxidation of charcoal and the formation of CO_2_ takes place [68]. Thus, it is believed that peaks at temperatures ranging from approximately 400–650 °C are derived not only from dehydroxylation processes, but also from the oxidation of charcoal originating from the decomposition of surfactants. The total losses in the mass of bentonites modified with HDTMA and TX100 and TX100 alone are different depending on the amount of surfactants adsorbed—the more the surfactants are adsorbed, the greater the loss of mass.

The analysis of curves for bentonite modified with HDTMA and TX100 and TX100 alone indicates that the amount of the nonionic surfactant used has a significant effect on the properties of the obtained hybrid material. A decrease in the amount of water molecules by replacing them with surfactant molecules improves the hydrophobic properties of modified bentonite. Furthermore, decomposition temperatures for all the modified bentonites are higher than that of pure surfactants, which suggests that compared with the neat surfactant, the thermal stability of the intercalated surfactant is greatly improved.

### 3.5. BET

**Figure 5:**
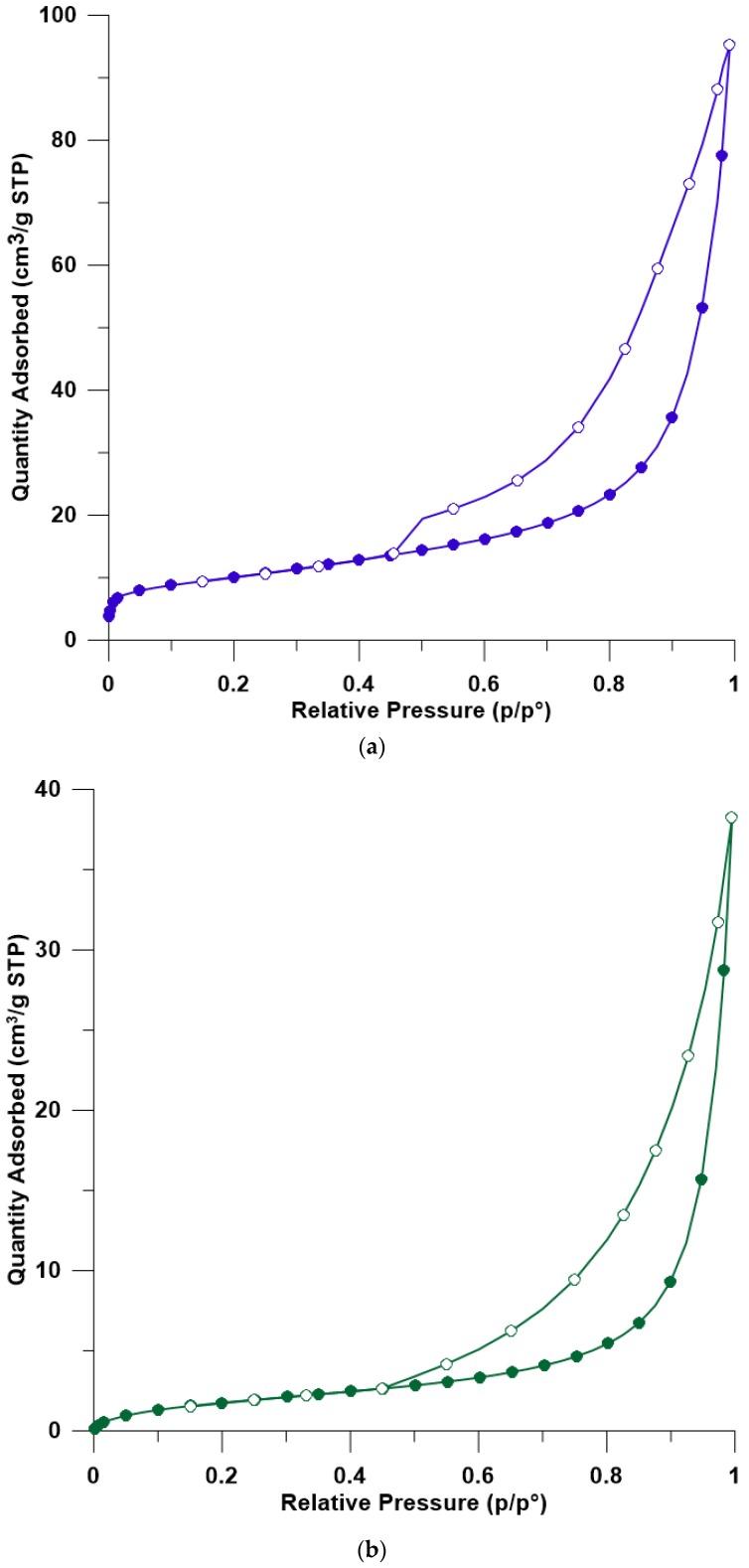

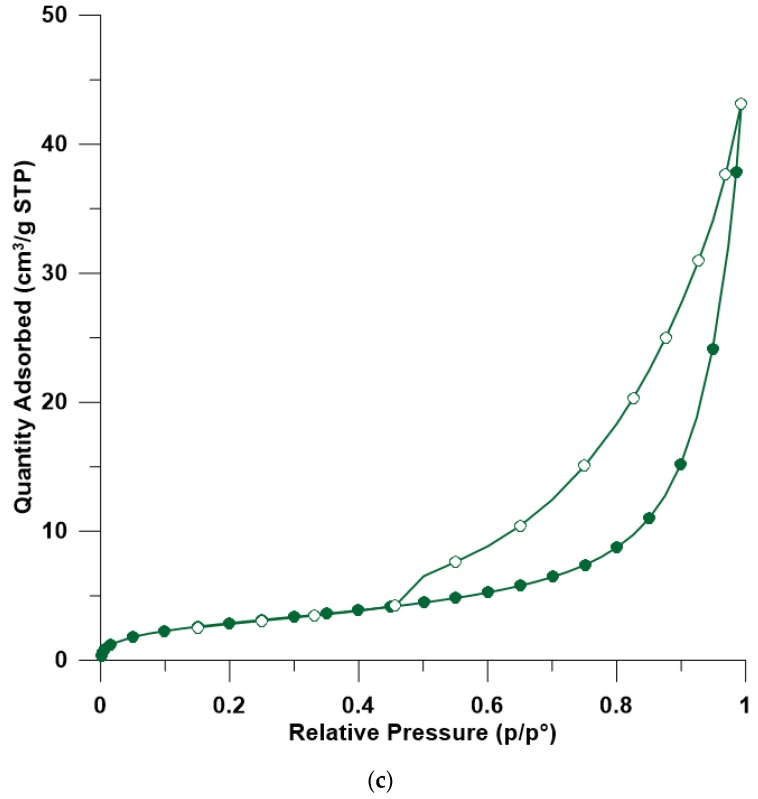
Comparison of N_2_ adsorption and desorption isotherms at −196 °C for: (**a**) BENT, (**b**) HD-TX-48, and (**c**) TX-48. Filled symbols—sorption of N_2_; unfilled symbols—desorption of N_2_.

Low-temperature nitrogen adsorption (N_2_-BET) tests provided information on the textural parameters of bentonite and organo-bentonite. The analysis was conducted only for few selected samples (BENT, HD-TX-48, and TX-48) due to their similarity in textural properties of surfactant-modified clay minerals. Based on the low-temperature adsorption and desorption of nitrogen, sorption/desorption isotherms were constructed (Figure 5). The IUPAC (International Union of Pure and Applied Chemistry) classification distinguishes I–VI types of adsorption isotherms. The shape of the isotherm of all sorbents corresponds to a type IV isotherm and H4 type of hysteresis loop, which are attributed to mesoporous materials with narrow slit-like pores [77]. Characteristic features of the type IV isotherm are its hysteresis loop, which is associated with capillary condensation taking place in mesopores, and the limiting uptake over a range of high p/p°. This type of isotherm also suggests that sorption is multilayered. The inflection points of the isotherms are not clear. This indicates that in the beginning, the monolayers and the formation of multilayers on the surface of the adsorbent occurred [78]. In the range of relative pressures below p/p° of approximately 0.45, micropores are filled, and above this value, meso- and macropores are filled. For all sorbents, hysteresis loops are visible, which are directly related to the capillary action of liquid nitrogen occurring in the mesopores [77].

The specific surface area of BET of organo-bentonites decreased significantly compared to natural bentonite. The modification also affected the total pore volume; however, the proportion of individual pore classes was maintained (Table 2). HD-TX-48 was characterized by the lowest BET surface area and total pore volume, which is associated with the modification process. Bentonite was first modified with the cationic surfactant and then with the nonionic surfactant. The resulting phase occupied more space, which was not observed for natural bentonite or bentonite modified with TX100 alone, thus preventing the probe from penetrating the pores in the bentonite. These results are consistent with the existing literature. In general, a higher loading of bentonite by surfactant molecules reduces the surface area and porosity of the modified sorbents [11,66,79].

### 3.6. SEM

**Figure 6:**
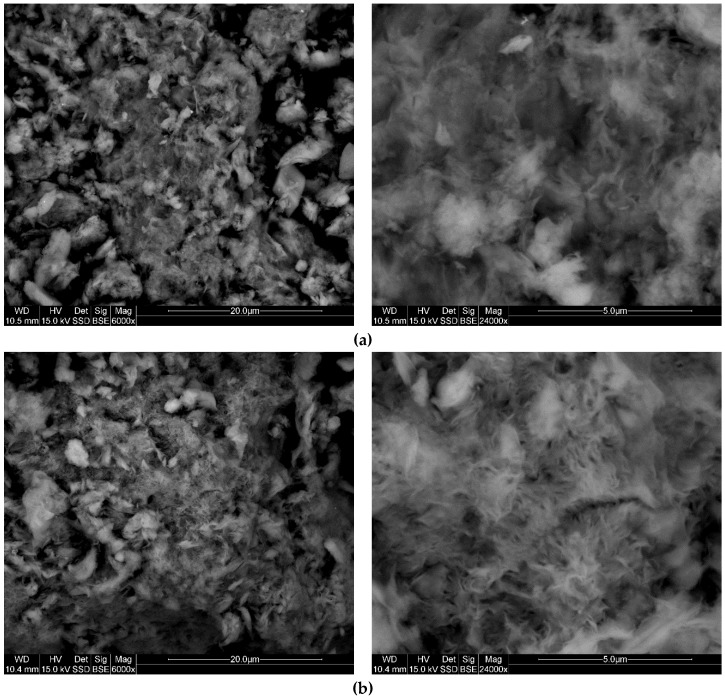

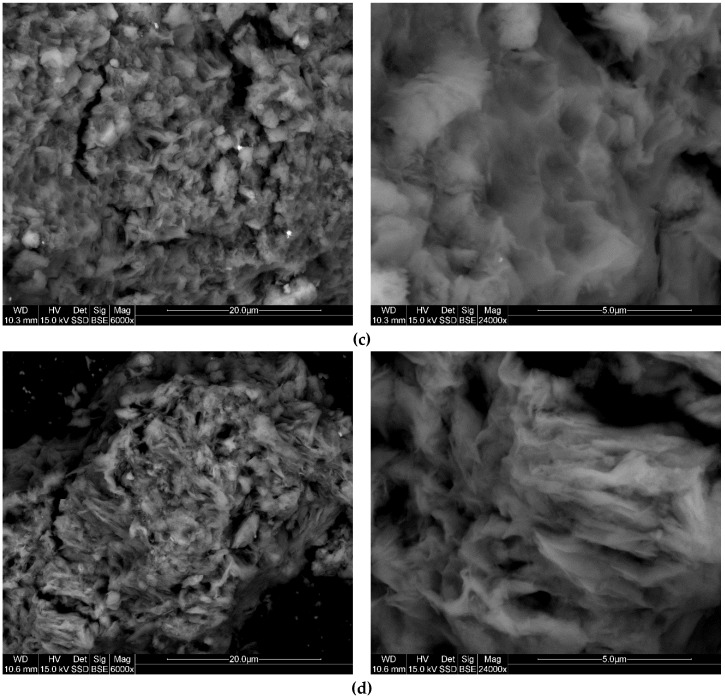
Scanning electron microscopy (SEM) images of samples: (**a**) BENT, (**b**) HD-B, (**c**) HD-TX-48, and (**d**) TX-48 (WD—working distance, HV—high voltage, Det—detector, Sig—signal, Mag—magnification, SSD—Single Shot Detector, BSE—Backscattered Electrons).

SEM was used to study the changes in morphology of bentonite upon intercalation of different surfactants (Figure 6). The analysis was conducted only for few selected samples (BENT, HD-B, HD-TX-48, and TX-48). Figure 6a represents natural bentonite. The characteristic smectite texture resembling a tissue layer is visible, however the separation zones are not distinct. The morphology of the sample after the modification with HDTMA did not change significantly; thus, the typical morphology of montmorillonite is evident (Figure 6b). It is caused by the modification type—HDTMA is mostly intercalated into the interlayer space of smectite. Further modification of HDTMA–bentonite with TX100 resulted in visible changes to the morphology of bentonite. Modification with TX100 takes place mostly on the surface of the sample; thus, the surface is coated with surfactant. It is also confirmed by previous results, especially BET. TX100 molecules are attached to the surface of HDTMA-modified bentonite and the disappearance of the tissue structure can be observed. Figure 6d represents the morphology of the bentonite modified with TX100 alone. The tissue-like texture remains, however some changes are visible. A layers-like texture is more oriented and organized than for natural bentonite [64]. It confirms that TX100 molecules are adsorbed mostly onto the surface.

## 4. Conclusions

In summary, this study investigates the structural characterization of bentonite modified with cationic and nonionic surfactants. The modification process of bentonite with HDTMA-Br and with TX100 was successful. According to the results, the presence of a cationic surfactant in the sample significantly enhanced the adsorption of nonionic surfactant molecules compared to natural bentonite. Pre-attached HDTMA molecules act as anchors on the bentonite surface, allowing the TX100 form mixed aggregates with HDTMA through chain–chain interactions. XRD analysis of the surfactant–bentonite systems revealed that nonionic surfactants cause slight expansion of the interlayer space of smectite, regardless of the presence of cationic surfactants. This suggests that the modification of bentonite with TX100 occurs mostly on the surface. Analysis of BET specific surface areas, FTIR spectra, SEM images and TG/DTA curves elucidated the structural changes in bentonite due to adsorption of surfactants and proved the effectiveness of the modification process. FTIR spectra showed absorption bands that indicate the presence of surfactants adsorbed by clay minerals. The presence of OH vibration bands in samples with adsorbed TX100 suggests interaction of surfactant molecules with the silicate through the functional groups of the surfactant and the water coordinated to the exchangeable cations of clay minerals by ion–dipole or hydrogen bonding. The analysis of TG/DTA curves indicates that intercalation of surfactants increased the structural and thermal stability of organo-bentonites. Moreover, as calculated by the nitrogen adsorption–desorption isotherms, the intercalation of surfactants decreased the specific surface area and the total pore volume of bentonite. SEM images confirmed that modification of bentonite with nonionic surfactants takes place mostly on the surface.

Although the modification of most of the clay minerals with HDTMA is well-described in the literature, there is still a lack of information regarding the use of nonionic surfactants such as Triton X-100. The results of this study contribute to the further understanding of the properties of clay and clay minerals modified with cationic and nonionic surfactants, as well as the determination of their possible ways of utilization. Surfactant-modified clay minerals are known as effective sorbents of both organic and inorganic compounds. New and less toxic hybrid materials based on nonionic surfactants can be an interesting alternative for the commonly used clay mineral-based sorbents.

## Figures and Tables

**Table 1:** The amount of Triton X-100 (TX100) adsorbed onto natural bentonite and bentonite with pre-adsorbed hexadecethyltrimethylammonium (HDTMA).

Initial Concentration of TX100 (mmol/100 g)	Amount of TX100 Adsorbed (mmol/100 g)	
	Bentonite with HDTMA Pre-Adsorbed	Natural Bentonite
2	1.96	1.74
6	5.08	4.66
12	11.94	11.08
24	22.30	23.72
48	43.24	32.68
60	59.30	29.24
80	73.70	31.62
120	108.92	34.50
160	124.72	39.96

**Table 2:** The textural parameters of BENT, HD-TX-48, and TX-48.

Sample	BET Surface Area (m^2^/g)	Total Pore Volume (cm^3^/g)	Volume of Micropores (cm^3^/g)	Volume of Mesopores (cm^3^/g)	Volume of Macropores (cm^3^/g)
BENT	35.7	0.143	0.015	0.098	0.03
HD-TX-48	7.1	0.053	0.002	0.032	0.019
TX-48	11.0	0.063	0.004	0.047	0.012
